# Socializing Models During Lactation Alter Colonic Mucosal Gene Expression and Fecal Microbiota of Growing Piglets

**DOI:** 10.3389/fmicb.2022.819011

**Published:** 2022-07-07

**Authors:** Yanju Bi, Haidong Wei, Haoyang Nian, Runze Liu, Wenbo Ji, Honggui Liu, Jun Bao

**Affiliations:** ^1^College of Animal Science and Technology, Northeast Agricultural University, Harbin, China; ^2^Key Laboratory of Swine Facilities Engineering, Ministry of Agriculture and Rural Affairs, Harbin, China

**Keywords:** social environment, short-chain fatty acids, RNA-seq, piglets, fecal microbiota

## Abstract

The enrichment of the social environment during lactation alleviates the stress of weaned piglets. It is significant to understand how the enriched social environment improves the weaning stress of piglets. RNA sequencing (RNA-seq) of colonic mucosa, 16S rRNA sequencing of feces, and short-chain fatty acids (SCFAs) of colonic content were used to determine the effects of social contact during lactation. In this study, thirty litter lactating piglets were divided into intermittent social contact (ISC) group that contacted with neighbors intermittently, continuous social contact (CSC) group that contacted with neighbors starting at day (D) 14 after birth, and control (CON) group in which piglets were kept in their original litter. The piglets were weaned at D35 and regrouped at D36. The colonic mucosal RNA-seq, fecal microbes, and SCFAs of colonic contents of 63-day-old piglets were analyzed. The results of RNA-seq showed that compared with the CON group, the pathways of digestion and absorption of minerals, protein, and vitamins of piglets were changed in the ISC group, whereas the pathways of retinol metabolism and nitrogen metabolism in the colonic mucosal were affected and stimulated the immune response in the CSC group. Compared with the CON group, the abundances of pernicious microorganisms (*Desulfovibrio, Pseudomonas, Brevundimonas*, etc.) in the CSC group and pernicious microorganisms (*Desulfovibrio, Neisseria, Sutterella*, etc.) and beneficial bacteria (*Bifidobacterium, Megamonas*, and *Prevotella_9*) in the ISC group were significantly higher (*p* < 0.05). The abundances of proinflammatory bacteria (*Coriobacteriaceae_unclassified, Coprococcus_3*, and *Ruminococcus_2*) in the CSC group were significantly increased (*p* < 0.05), but the abundances of SCFAs producing bacteria (*Lachnospiraceae_UCG-010, Parabacteroides, Anaerotruncus*, etc.) and those of anti-inflammatory bacteria (*Eubacterium, Parabacteroides, Ruminiclostridium_9*, and *Alloprevotella*) were significantly reduced (*p* < 0.05) in the CSC group. Compared with the CON group, the concentrations of microbial metabolites, acetate, and propionate in the colonic contents were reduced (*p* < 0.05) in the ISC group, whereas the concentration of acetate was reduced (*p* < 0.05) in the CSC group. Therefore, both ISC and CSC during lactation affected the composition of fecal microbes and changed the expression of intestinal mucosal genes related to nutrient metabolism and absorption of piglets.

## Introduction

Pigs are social animals, and sows lead their piglets back to the group at postpartum 12–14 days. The piglets are weaned at 14–17 weeks of age under natural conditions (Jensen, 1986). The piglets often share social interaction with individuals from other litters in early life (Graves, 1984). To improve economic efficiency, the piglets are weaned earlier at around 4 weeks of age (D'Eath and Lawrence, 2004). Piglets are exposed to new environments, foods, and groups at a younger age, leading to the production problems, such as transient anorexia, diarrhea, intestinal inflammation, intestinal microbial disturbance, and slow weight gain (Hameister et al., 2010; Campbell et al., 2013; Pohl et al., 2017; Meng et al., 2020). Thus, intensive farming damages the health and welfare of piglets. In the recent years, people paid more attention to the welfare of piglets. An enriched social environment during lactation reduces the fighting behavior of piglets and quickly forms an establishment of hierarchy during the weaned regrouping (D'Eath, 2005; Kutzer et al., 2009), improving the adaptability of piglets to the new environment (Kutzer et al., 2009; Ko et al., 2020). In addition, enriched social contact in early life reduces skin damage, and the content of stress biomarkers and increases the play behavior of weaned piglets (Salazar et al., 2018; Ko et al., 2020). Our recent study also showed that the lactating enriched social experience reduced the cortisol level and feed conversion ratio (FCR) of piglets during regrouping and increased the daily gain weight (Ji et al., 2021). Thus, enriched social contact during lactation is beneficial for the growth and welfare of piglets.

Social contact mediates the acquisition and exchange of microbial symbiotes (Wikberg et al., 2020). The formation and maintenance of a healthy gut microorganism in early life are critical for piglets because the changes in early microorganisms can affect the composition of the microbial community, which affects the health status and growth performance of pigs in later life (Guevarra et al., 2019). Intestinal microbes affect the physiological immunity, behavior, and nutrient metabolism of the host (Danneskiold-Samsoe et al., 2019). Furthermore, intestinal microbes in the early stages of animal life are not stable and are affected by the environment, nutrition, and genetics, which have long-term effects on the colonization of host microorganisms (Eisenstein, 2020). Social contact affects the intestinal microbial composition of rhesus monkeys and ponies (Antwis et al., 2018; Trosvik et al., 2018). Intestinal microbes are associated with intestinal development, diarrhea, feed efficiency, and growth performance of piglets (Tang et al., 2020). For example, *Prevotella_9* was negatively correlated with the diarrhea rate of piglets (Hung et al., 2019), and *Prevotella_2* was beneficial for feed intake, feed efficiency, fat accumulation, and muscle growth of newborn piglets (Qi et al., 2021). Thus, a healthy gut microbe in early life is important for the health and performance of piglets. Short-chain fatty acids (SCFAs) are mainly produced by microbial fermentation of undigested dietary carbohydrates (Hamer et al., 2008). They maintain intestinal homeostasis, provide energy for intestinal epithelial cells, and prevent intestinal inflammation and oxidative stress (Dalile et al., 2019). The concentration of SCFAs in the colon is affected by the substrate flowing into the large intestine, the size of the organ, and the abundance and composition of bacteria in the lumen of pig (Haenen et al., 2013). Saladrigas-García et al. (2021)) showed that the enriched social contact during lactation affected the microbial composition of cecal content, such as *Lactobacillaceae* and *Fusobacteriaceae*, and downregulated toll-like receptor 2 (TLR-2) gene expression of jejunum in weaned piglets. We speculated that social contact during lactation affected the composition of fecal microbes, which might affect the colonic contents of SCFAs and regulate the colonic gene expression of piglets at the end of the growing period. This study will provide new insights into the lactating contact models of strong adaptability to the new environment for alleviating the weaning stress in piglets.

## Materials and Methods

### Ethics Statement

All procedures described in this study had been approved by the Animal Care and Use Committee of Northeast Agricultural University [NEAU-(2011)-9].

### Animal Management and Sampling

About thirty litter crossbred piglets (Large White × Duroc × Min-pig) were selected from the Acheng commercial pig farm in Harbin, Heilongjiang Province, China. The lactation period was D0-35, and the growing period was D36-63. The sows were moved into the farrowing pens before 1 week of parturition. The area of delivery was 2.20 × 3.00 m, and an activity area of 0.80 × 1.60 m was provided for piglets running. The details of pen size were described in our previous study (Ji et al., 2021). The 30 litter piglets were divided into three groups: control (CON), intermittent social contact (ISC), and continuous social contact (CSC) groups. CON group: six sows and their offspring were raised in the farrowing pens from birth to D35, and there was no chance to get in contact with the strangers. These six litter piglets were regrouped into six growing pens with 10 individuals with body weight of 9.42 ± 0.17 kg and gender (the male were castrated at D7) at D36, and the growing pen size was 6.12 m^2^; ISC group: 12 sows and their offspring were raised in the farrowing pens from birth to D35. The wooden door between the adjacent pens was removed from 1,300 to 1,600 at D14, D15, D21, D22, D28, D29, D33, and D34, and the piglets socialized freely. These 12 litter piglets were regrouped into six pens with 10 individuals with a body weight of 9.24 ± 0.39 kg and gender at D36; CSC group: 12 sows and their offspring were raised in the farrowing pens from birth to D35, and the wooden door between the adjacent pens was removed at D14. The piglets socialized from D14 to D35, and these 12 litter piglets were regrouped into six pens with 10 individuals with a body weight of 9.60 ± 0.42 kg and gender at D36. Every two litters were regarded as a unit of social contact during lactation in ISC and CSC groups. The rearing environment was maintained as described previously (Ji et al., 2021), and the composition of the feed is shown in Table 1. Piglets were given *ad libitum* to water. All nutrients met or exceeded National Research Council (NRC) (2012) recommendations. The fresh rectal feces of female piglets were collected by sterile cotton at D63, and 24 samples, including 6 ISC, 8 CSC, and 10 CON samples, were selected as valid samples. One piglet from each litter was euthanized by intra-arterial injection of 200 mg/kg pentobarbital sodium (Sigma-Aldrich, St Louis, MO, USA) for collecting colonic mucosa and colonic contents at D63. The colonic mucosa was rinsed with physiological saline. The rectal feces, colonic mucosa, and content samples were frozen immediately in liquid nitrogen and then stored at −80°C for further analysis.

**Table 1:** Diet formula of lactating-growing piglets.

**Item ingredients**	**Creep feed**	**Nursery diet**
Crude protein (%)	≥20.00	≥16.50
Crude fiber (%)	≤4.00	≤6.00
Crude ash (%)	≤10.00	≤7.00
Calcium (%)	0.60–1.00	0.50–1.20
Total phosphorus (%)	≥0.30	≥.45
Sodium chloride (%)	0.30–1.50	0.30–1.20
Water (%)	≤14.00	≤14.00
Lysine (%)	≥1.50	≥1.20

### RNA Sequencing Analysis

Total RNAs were isolated and purified from colonic mucosa using TRIzol (Invitrogen, CA, USA) and quantified by NanoDrop ND-1000 (NanoDrop, Wilmington, DE, USA). Then, the RNA integrity was analyzed by Bioanalyzer 2100 (Agilent, CA, USA). The mRNA with polyadenylic acid (PolyA) was isolated through two-round purification using Dynabeads Oligo (dT) 25-61005 (Thermo Fisher, CA, USA). The captured mRNA was fragmented into small pieces using a Magnesium RNA Fragmentation Module (NEB cat.e6150S, USA) at 94°C for 5–7 min, and the cleaved RNA fragments were then reverse-transcribed to create the complementary DNA (cDNA) by SuperScript™ II Reverse Transcriptase (Invitrogen, cat. 1896649, USA). The cDNA library was constructed, and double-ended sequencing was performed using Illumina Novaseq™ 6000 (LC-Bio Technology CO., Ltd., Hangzhou, China) to obtain a 150 bp peer end sequence. A total of three piglets from each experimental group were analyzed by RNA-seq. Using HISAT after preprocessing of the effective data (Valid Data) to map the reference genome (ftp://ftp.ensembl.org/pub/release-96/fasta/sus_scrofa/dna/), StringTie (http://ccb.jhu.edu/software/stringtie/), and Ballgown were used to estimate the gene expression level of each sample. The reliability of biological repeatable sequencing depth was calculated by packageversion 1.34.0. The statistical power was 0.98, which was calculated using RNASeqPower (https://doi.org/10.18129/B9.bioc.RNASeqPower). The gene expression level was estimated by calculating the number of base exon fragments per million mapped (FPKM) reads. The difference multiple log_2_ (fold-change) >1 or log_2_ (fold-change) <-1 and *p* < 0.05 were defined as differentially expressed genes (DEGs) for further analysis. Kyoto Encyclopedia of Genes and Genomes (KEGG) pathway analysis was performed using the R software package to analyze the enrichment of DEGs in certain KEGG pathways. Benjamini–Hochberg method was used to reduce the false discovery rate (FDR) with a cutoff of 0.05 for identifying differential KEGG pathways. The RNA-seq's Submission ID was SUB10435518, and BioProject ID was PRJNA771754.

### Quantitative Real-Time Polymerase Chain Reaction Validation of DEGs

Total RNAs were extracted from the colonic mucosa according to the instructions for the RNAiso Plus Kit (Takara, Dalian, China). RNA integrity and quality were measured by 1% agarose electrophoresis, and RNA concentration and purity were measured at 260/280 nm (Gene Quant 130/100, United States). According to RR407 (Takara, Dalian, China) reagent instructions, cDNA was synthesized. The synthesized cDNA was stored at −80°C until use. Real-time fluorescence quantitative PCR (qPCR) was performed using LightCycler 480 (Roche, Switzerland). The reaction mixture was 10 μl, which consisted of 1 μl cDNA, 0.3 μl upstream primers, 0.3 μl downstream primers, 3.4 μl PCR-grade water, and 5 μl 2X Roche Fast Universal SYBR Green Master kit (no. 04913914001, Roche, Switzerland). The qPCR program was as follows: 95°C for 10 min, 40 cycles, 95°C for 15 s, 60°C for 1 min, and 60°C for 20 s. The dissolution curve showed a peak for each PCR product. The method of 2^−ΔΔCt^ was used to calculate the relative expression of each gene mRNA. The chemokine (C-C motif) ligand 17 (*CCL17*), chemokine (C-C motif) ligand 26 (*CCL26*), oncostatin M (*OSM*), chemokine (C–C motif) ligand 12 (*CCL2*), and arginine vasopressin (*AVP*) genes were used to verify the transcriptome results of the CSC and CON groups, and interferon-gamma (*IFNG*), *OSM*, peptide YY (*PYY*), *CCL17*, and retinol dehydrogenase 5 (*RDH5*) genes were used to verify the transcriptome results of ISC and CON groups. β-actin was used as the internal reference gene to quantify the expression of target DEGs. The specific base pairs of each gene are shown in Table 2.

**Table 2:** Gene sequences used for real-time fluorescence quantitative PCR analysis.

**Gene**	**Reference sequence**	**Primer sequence (5^**′**^-3^**′**^)**
CCL17	NM_001256147.2	Forward: GAGTGCTGCCTGGAGTACTTCAAAG Reserve: GTGTCCTTGGGGTCAGAACAGATG
CCL26	NM_001078665.2	Forward: TATCCTGAGTGTCCACCTCGGAAC Reserve: GGCAGGATCTTGTGGCTGTATTGG
OSM	XM_001929161.4	Forward: GCTCAGGCAAATACCACGAACTCC Reserve: GATCCTTGCAGTGCTCCTTCAGTC
CCL2	NM_214214.1	Forward: CAGTCACCTGCTGCTATACACTTACC Reserve: TCACTGCTTCTTTAGGACACTTGCTG
AVP	NM_213952.2	Forward: AGAGGGCCATGTCCGACTTG Reserve: CAGATACTGGGCCCGAAGCA
PYY	NM_001256528.1	Forward: CCACTACCTCAACCTGGTCACTCG Reserve: GGAAGAGCAGTTTGGAGAGAAGAGC
RDH5	XM_013997618.2	Forward: GAGGCTACTGCGTCTCCAAGTTTG Reserve: TTTCCGGGTTTGTTATAGGGGTTTGG
β-actin	XM_021086047.1	Forward: GGCACCACACCTTCTACAACGAG Reserve: TCATCTTCTCACGGTTGGCTTTGG

*CCL, chemokine (C-C motif) ligand; OSM, oncostatin M; AVP, arginine vasopressin; IFNG, interferon gamma; PYY, peptide YY; RDH5, retinol dehydrogenase 5*.

### 16S rRNA Gene Sequencing

The E.Z.N.A.® Fecal DNA Kit (D4015, Omega, Inc., Norcross, GA, USA) was used to extract the fecal DNA of piglets, and the DNA was purified by AMPure XT beads (Beckman Coulter Genomics, Danvers, MA, USA) according to the manufacturer's instructions. The V3–V4 region of the 16S rRNA gene was amplified using special primers (forward primer 341F: CCTACGGGNGGCWGCAG; reverse primer 805R: GACTACHVGGGTATCTAATCC) and quantified by Qubit (Invitrogen, Carlsbad, CA, USA). The amplicons were prepared for sequencing, and the size and quantity of the amplicon library were evaluated by Agilent 2100 Bioanalyzer (Agilent, Santa Clara, CA, USA) and Illumina Library Quantitative Kit (No.KK4824, Kapa Biosciences, Woburn, MA, USA). The library was sequenced on the NovaSeq PE250 platform.

The 16S rRNA gene sequence was processed on the Illumina NovaSeq platform according to the manufacturer's recommendations (LC-Bio, Hangzhou, China). The paired-end reads were used FLASH to merge. According to fqtrim (version 0.94), quality filtering was performed on the original read data to obtain high-quality sequences. Alpha diversity and beta diversity were calculated after normalizing them to the same sequences. According to the SILVA (release 132) classifier, the feature abundance was normalized to the relative abundance of each sample. Alpha diversity (Chao1, observed species, Shannon, and Simpson) and principal coordinate analysis were applied for analyzing the complexity of species diversity by Quantitative Insights into Microbial Ecology 2 (QIME2) and were plotted by the R package (v3.5.2). The fecal microbiome's submission ID was SUB8294724 and SUB10769336, and BioProject IDs were PRJNA668937 and PRJNA786659.

### Interactions Analysis Between Colonic Mucosal Gene and Microbiome

Digitally expressed genes from colonic mucosa (9 samples, with 3 samples in each treatment) and fecal microbial taxa (24 samples, including 6 ISC, 8 CSC, and 10 CON samples) were used. In the ISC and CON groups, we further subset DEGs in the ISC vs. CON enriched in the differential KEGG pathways, leaving 49 genes for downstream analysis. There were 42 differential microbial taxa between ISC and CON groups with an absolute expression log ratio >1 and *p* < 0.05 for further processing. Then, we performed a correlation analysis between 49 DEGs of the colonic mucosa and 37 taxa (genus level) of feces. In the CSC and CON groups, we further subset DEGs in the CSC vs. CON enriched in the differential KEGG pathways, leaving 42 genes for downstream analysis. Then, 58 differential microbial taxa were found in CSC and CON groups with an absolute expression log ratio >1 and *p* < 0.05. Then, we performed a correlation analysis between 42 DEGs of the colonic mucosa and 58 taxa (genus level) of feces. Spearman's correlation was used for this analysis (Weiss et al., 2016). The significance of the correlation was indicated using an asterisk.

### Colonic Content SCFAs Analysis

The SCFAs (acetic acid, propionic acid, isobutyric acid, butyric acid, isovaleric acid, and valeric acid) in colonic contents of piglets from three groups were determined by the gas chromatography-mass spectrometry (GC-MS) analysis method. The 50 mg sample was homogenized in the mixed solution (50 μl of 15% phosphoric acid, 100 μl of 125 μg/ml internal standard (isocaproic acid) solution and 400 μl of ether) and centrifuged at 4°C for 10 min at 12,000 rpm. The supernatant was then analyzed by GS-MS (Thermo TRACE 1310-ISQ LT, USA). The chromatographic column Agilent HP-InnoWax column was used in this study. In the operation process of GS-MS, the initial temperature was 90°C. The temperature was heated to 120°C at 10°C/min, then to 150°C at 5°C/min, and finally to 250°C at 25°C/min for 2 min. The temperature of the inlet was 250°C. SCFAs were identified by the retention time of standard compounds.

### Statistical Analysis

The data of qRT-PCR validation and SCFAs levels were analyzed by Statistical Package for the Social Sciences (SPSS) (IBM-SPSS Inc., Chicago, IL, USA). All data were tested for normal distribution using the Kolmogorov–Smirnov test. The significance of the difference in ISC vs. CON and CSC vs. CON was analyzed by Student's *t*-test. If *p* < 0.05, the differences were considered statistically significant. The experimental results were expressed as mean ± standard error of the mean (SEM). In addition, the differential abundant microbial genera in ISC group vs. CON group and CSC group vs. CON group were identified as |log_2_(fold-change)| > 1, and the *p*-value was calculated by the Wilcox test.

## Results

### RNA-Seq Analysis

**Figure 1:**
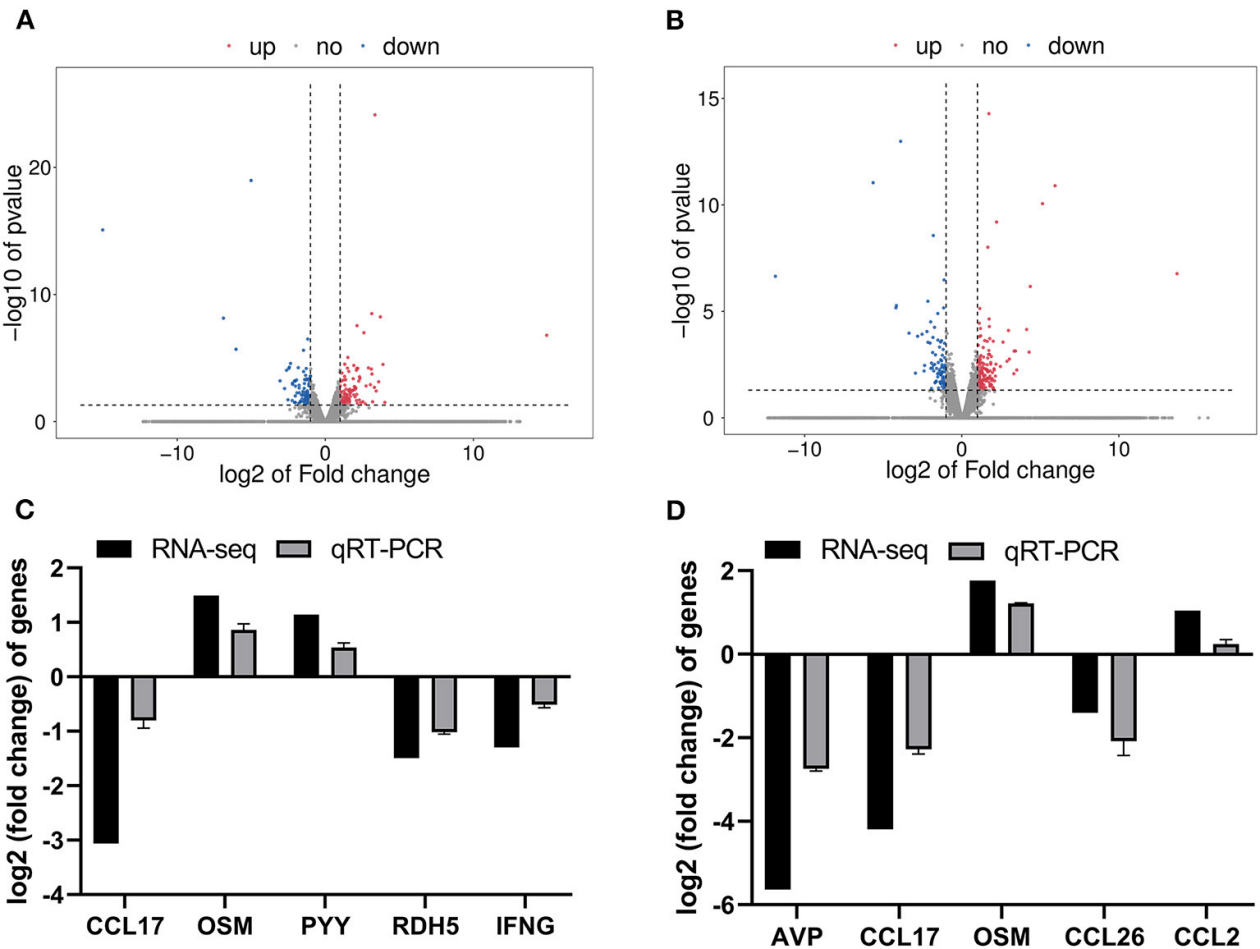
The RNA-seq analysis of different social contact patterns in the colonic mucosa of piglets. **(A)** Volcano plot of DEGs between ISC and CON groups. **(B)** Volcano plot of DEGs between CSC and CON groups. **(C)** The relative gene expression levels and transcriptional FPKM between ISC and CON group. **(D)** The relative gene expression levels and transcriptional FPKM between CSC and CON group. Gene expression at mRNA level was calculated by RT-PCR. The RT-PCR results were analyzed by Student's *t*-test and the results were represented as mean ± SEM (*n* = 3). ISC, intermittent social contact; CSC, continuous social contact; CON, control; FPKM, base exon fragments per million mapped.

A total of 9 cDNA libraries contained an average of 46,648,961 raw data reads, and the average of 45,475,308 valid reads remained after the removal of sequencing joints and low-quality sequencing data (Supplementary Table 1). In this study, DEGs were identified as *p* < 0.05 and |log_2_(fold-change)| > 1. Distributions of DEGs of ISC group vs. CON group and CSC group vs. CON group are shown in Figures 1A,B. The blue dots represented the downregulated genes, and the red dots represented the upregulated genes in the volcanic map. Compared with the CON group, there were 215 DEGs in the ISC group containing 113 upregulated and 102 downregulated DEGs (Figure 1A) and 245 DEGs in the CSC group containing 157 upregulated and 88 downregulated DEGs (Figure 1B). The specific DEGs of ISC vs. CON and CSC vs. CON are shown in Supplementary Tables 2, 3, respectively.

### KEGG Pathway Analysis of DEGs

Compared with the CON group, 22 differential pathways were identified in the ISC group (Table 3). A total of six pathways were enriched in nutrient absorption, including vitamin digestion and absorption, mineral absorption, saliva secretion, carbohydrate digestion and absorption, protein digestion and absorption, and pancreatic secretion. Besides, two pathways were related to the endocrine system, including renin secretion and the relaxin signaling pathway. Peroxisome proliferator-activated receptor (PPAR) signaling pathway and retinol metabolism were related to nutrition metabolism. There were 20 different pathways between the CSC and CON groups (Table 4). A total of thirteen pathways were related to nutrient metabolism, including retinol metabolism, metabolism of xenobiotics by cytochrome P450, synthesis and degradation of ketone bodies, nitrogen metabolism, drug metabolism by cytochrome P450, steroid hormone biosynthesis, ascorbate and aldarate metabolism, arginine biosynthesis, pentose and glucuronate interconversions, terpenoid backbone biosynthesis, arachidonic acid metabolism, and butanoate metabolism. In addition, two pathways related to digestive enzyme secretion were pancreatic secretion and proximal tubule bicarbonate reclamation.

**Table 3:** KEGG pathway enrichment of DEGs (ISC vs. CON).

**Pathway ID**	**Pathway name**	**Upregulated genes**	**Downregulated genes**	***p*-value**
ko04924	Renin secretion	KCNMA1, ADORA1, ENSSSCG00000033041	ENSSSCG00000006932, PLCB1	0.002
ko04977	Vitamin digestion and absorption	CUBN, APOA4	CBLIF	0.003
ko05142	Chagas disease (American trypanosomiasis)	TGFBR1, ENSSSCG00000033041	PLCB1, CCL3L1, IFNG	0.009
ko04978	Mineral absorption	SLC6A19	MT1A, MT2A	0.010
ko04390	Hippo signaling pathway	TGFBR1, AREG, FGF1, FZD8	FRMD1, PPP1CB	0.011
ko03320	PPAR signaling pathway	MMP3, APOC3, FABP6, ADIPOQ		0.012
ko04060	Cytokine-cytokine receptor interaction	TGFBR1, IL1RL1, OSM, GDF15	IL7R, CCL3L1, CCL17, IFNG	0.012
ko04970	Salivary secretion	KCNMA1, ENSSSCG00000033041, ENSSSCG00000033452	PLCB1	0.013
ko04973	Carbohydrate digestion and absorption	ENSSSCG00000015691, ENSSSCG00000030013	ENSSSCG00000028277	0.014
ko04974	Protein digestion and absorption	SLC15A1, SLC6A19	COL11A2, ENSSSCG00000016245	0.016
ko04657	IL-17 signaling pathway	MMP3, ENSSSCG00000033452	CCL17, IFNG	0.019
ko04926	Relaxin signaling pathway	TGFBR1, MMP3, ENSSSCG00000033041	PLCB1, ENSSSCG00000016245	0.020
ko04270	Vascular smooth muscle contraction	KCNMA1, ENSSSCG00000033041, ENSSSCG00000035649	PLCB1, PPP1CB	0.022
ko05146	Amoebiasis	ENSSSCG00000033041	PLCB1, ENSSSCG00000016245, IFNG	0.024
ko00830	Retinol metabolism	HSD17B6	RDH5, ENSSSCG00000022724	0.025
ko04972	Pancreatic secretion	KCNMA1, ENSSSCG00000033041	ENSSSCG00000006932, PLCB1	0.026
ko04261	Adrenergic signaling in cardiomyocytes	PPP1R1A, SCN4B, ENSSSCG00000033041	PLCB1, PPP1CB,	0.027
ko04750	Inflammatory mediator regulation of TRP channels	ENSSSCG00000033041, P2RY2	PLCB1, PPP1CB	0.032
ko04080	Neuroactive ligand-receptor interaction	CHRNB4, TRH, ADORA1, PYY, GABRB2, ENSSSCG00000035649, P2RY2	NMU	0.044
ko04720	Long-term potentiation	PPP1R1A	PLCB1, PPP1CB	0.044
ko05332	Graft-versus-host disease	GZMB	IFNG	0.046
ko00290	Valine, leucine and isoleucine biosynthesis	ENSSSCG00000036865		0.050

*n = 3. DEGs, differentially expressed genes; ISC, intermittent social contact; CON, control*.

**Table 4:** KEGG pathway enrichment of DEGs (CSC vs. CON).

**Pathway ID**	**Pathway name**	**Upregulated genes**	**Downregulated genes**	***p*-value**
ko00830	Retinol metabolism	CYP4A24, CYP26B1, ENSSSCG00000040980	RDH5, CYP1A1, CYP2A19, ENSSSCG00000022724	<0.001
ko04270	Vascular smooth muscle contraction	CYP4A24, ACTG2, KCNMA1, KCNMB1, PLA2G2D	ADRA1D, AVP, PPP1CB	<0.001
ko00980	Metabolism of xenobiotics by cytochrome P450	ENSSSCG00000040980	CYP1A1, GSTA2, CBR2, ENSSSCG00000022724	<0.001
ko04060	Cytokine-cytokine receptor interaction	TGFBR1, TNFSF11, OSM, CXCR5, CCL2, GDF5, ENSSSCG00000036445, CCL26	TNFRSF8, CCL17, TNFRSF13C	0.001
ko04640	Hematopoietic cell lineage	CD22, CD19, MS4A1, FCER2	ANPEP	0.005
ko05204	Chemical carcinogenesis	ENSSSCG00000040980	CYP1A1, GSTA2, ENSSSCG00000022724	0.006
ko00072	Synthesis and degradation of ketone bodies	HMGCL, HMGCS2		0.007
ko00910	Nitrogen metabolism	CA4	CA2	0.020
ko00982	Drug metabolism - cytochrome P450	ENSSSCG00000040980	GSTA2, ENSSSCG00000022724	0.022
ko00140	Steroid hormone biosynthesis	ENSSSCG00000040980	CYP1A1, ENSSSCG00000022724	0.022
ko00053	Ascorbate and aldarate metabolism	ENSSSCG00000040980	ENSSSCG00000022724	0.023
ko00220	Arginine biosynthesis	ASS1, NOS2		0.028
ko00040	Pentose and glucuronate interconversions	ENSSSCG00000040980	ENSSSCG00000022724	0.034
ko00900	Terpenoid backbone biosynthesis	ICMT, HMGCS2		0.034
ko04964	Proximal tubule bicarbonate reclamation	CA4	CA2	0.034
ko04972	Pancreatic secretion	KCNMA1, SCTR, PLA2G2D	CA2	0.042
ko04933	AGE-RAGE signaling pathway in diabetic complications	TGFBR1, EGR1, CCL2	ENSSSCG00000016245	0.042
ko00983	Drug metabolism-other enzymes	ENSSSCG00000040980	GSTA2, ENSSSCG00000022724	0.043
ko00590	Arachidonic acid metabolism	CYP4A24, PLA2G2D	CBR2	0.043
ko00650	Butanoate metabolism	HMGCL, HMGCS2		0.044

*n = 3. DEGs, differentially expressed genes; CSC, continuous social contact; CON, control*.

### qRT-PCR Verification of DEGs

The results of transcriptional FPKM analysis and the relative mRNA expression of DEGs by qRT-PCR verification are shown in Figure 1. A total of five DEGs were selected to verify the RNA-seq data of the ISC group vs. CON group and CSC group vs. CON group by qRT-PCR. Compared with the CON group, the mRNA expression levels of *CCL17, RDH5*, and *IFNG* in the ISC group were downregulated, and the mRNA expression levels of *OSM* and *PYY* were upregulated (Figure 1C). The mRNA expression levels of *AVP, CCL17*, and *CCL26* in the CSC group were downregulated, and *OSM* and *CCL2* expression levels were upregulated (Figure 1D). The expression levels of these DEGs were consistent with the RNA-seq results.

### Analysis of Fecal Microbiome

A total of 24 valid fecal samples of piglets from three groups were obtained, with an average of 69,755 high-quality sequences per sample (Supplementary Table 4) after the screening, including 6 biological replicates in the ISC group, 8 replicates in the CSC group, and 10 replicates in the CON group.

### Analysis of Bacterial Diversity

**Figure 2:**
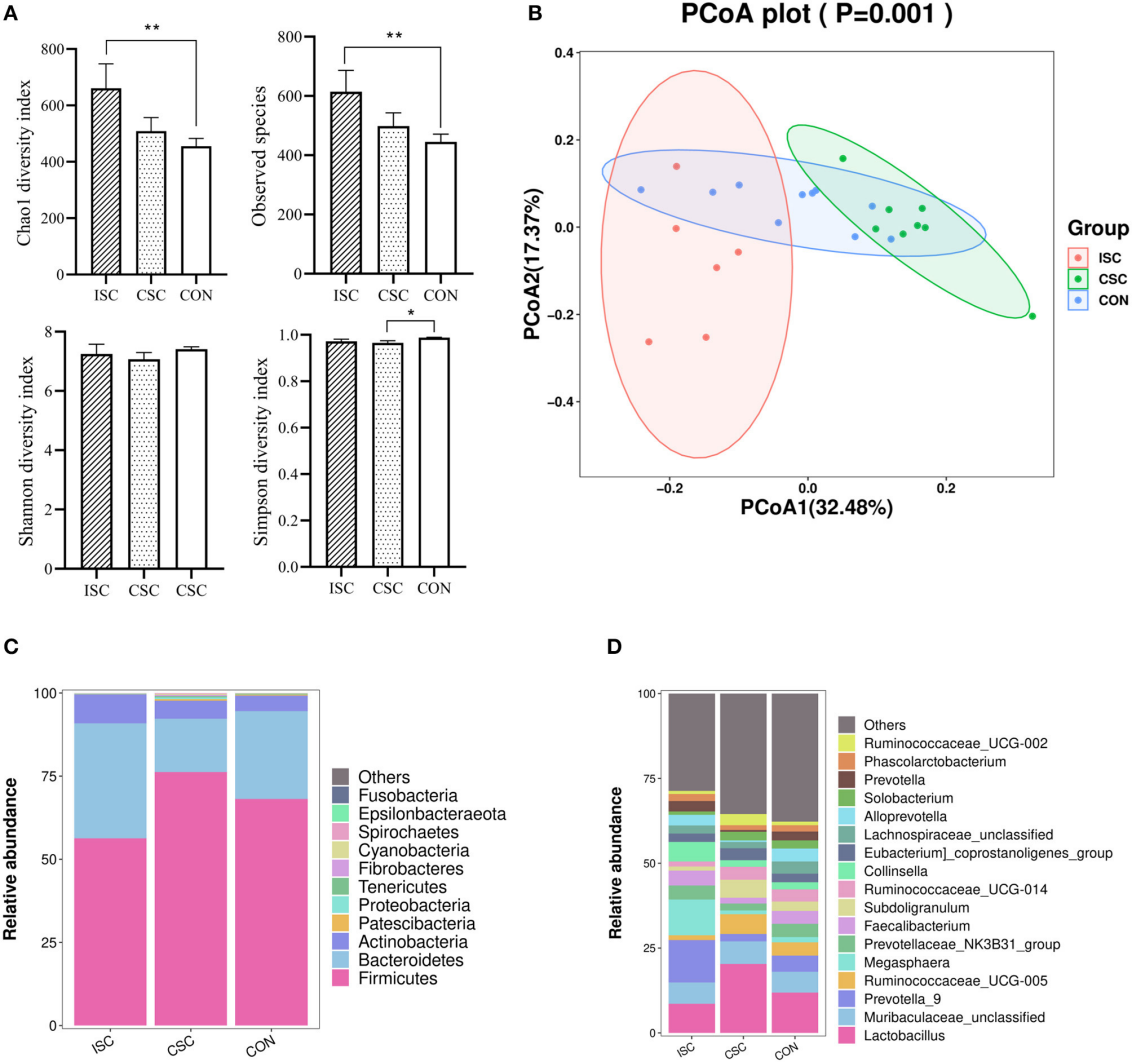
The fecal microbial diversity and taxa in piglets. **(A)** Alpha diversity of the fecal microbiota containing Chao1, observed species, Shannon, and Simpson. *p < 0.05, **p < 0.01; **(B)** principal coordinate analysis (PCoA, Bray–Curtis distance) of the fecal microbes. **(C)** Fecal microbial composition at the phylum level. **(D)** Fecal microbial composition at the genus level. ISC, intermittent social contact; CSC continuous social contact; CON, control.

Compared with the CON group, Chao1 index and observed species in the ISC group were significantly increased (*p* < 0.05), but those in the CSC group were not significantly different (*p* > 0.05). Simpson index was not significantly different in the ISC group but significantly decreased in the CSC group (*p* < 0.05) compared with the CON group. There was no significant difference in Shannon index between the ISC and CON groups and the CSC and CON groups (Figure 2A). Figure 2B demonstrated that the fecal microbial communities in both ISC and CSC groups were separated from the CON group, respectively.

### Comparison of Microbial Community

At the phylum level (Figure 2C), the microorganisms in the ISC group mainly comprised Firmicutes (56.32 ± 2.95%), Bacteroidetes (34.55 ± 2.53%), Actinobacteria (8.73 ± 7.91%), and Proteobacteria (0.14 ± 1.03%). The microorganisms in the CSC group were primarily composed of Firmicutes (76.22 ± 2.29%), Bacteroidetes (16.00 ± 1.99%), Actinobacteria (5.47 ± 0.40%), and Proteobacteria (0.53 ± 3.14%). In the CON group, Firmicutes (68.15 ± 2.64%), Bacteroidetes (26.44 ± 2.94%), Actinobacteria (4.64 ± 0.51%), and Proteobacteria (0.10 ± 1.04%) were the main bacteria. At the genus level (Figure 2D), the main microorganisms in the ISC group were *Prevotella_9* (12.50 ± 2.92%), *Megasphaera* (10.54 ± 5.34%), and *Lactobacillus* (8.60 ± 1.47%). The microorganisms in the CSC group were mainly composed of *Lactobacillus* (20.36 ± 4.46%), *Muribaculaceae_unclassified* (6.63 ± 1.47%), and *Ruminococcaceae_UCG-005* (5.84 ± 0.92%). CON group mainly consisted of *Lactobacillus* (11.88 ± 2.03%), *Muribaculaceae_unclassified* (6.14 ± 0.59%), and *Prevotella_9* (4.81 ± 1.15%).

**Figure 3:**
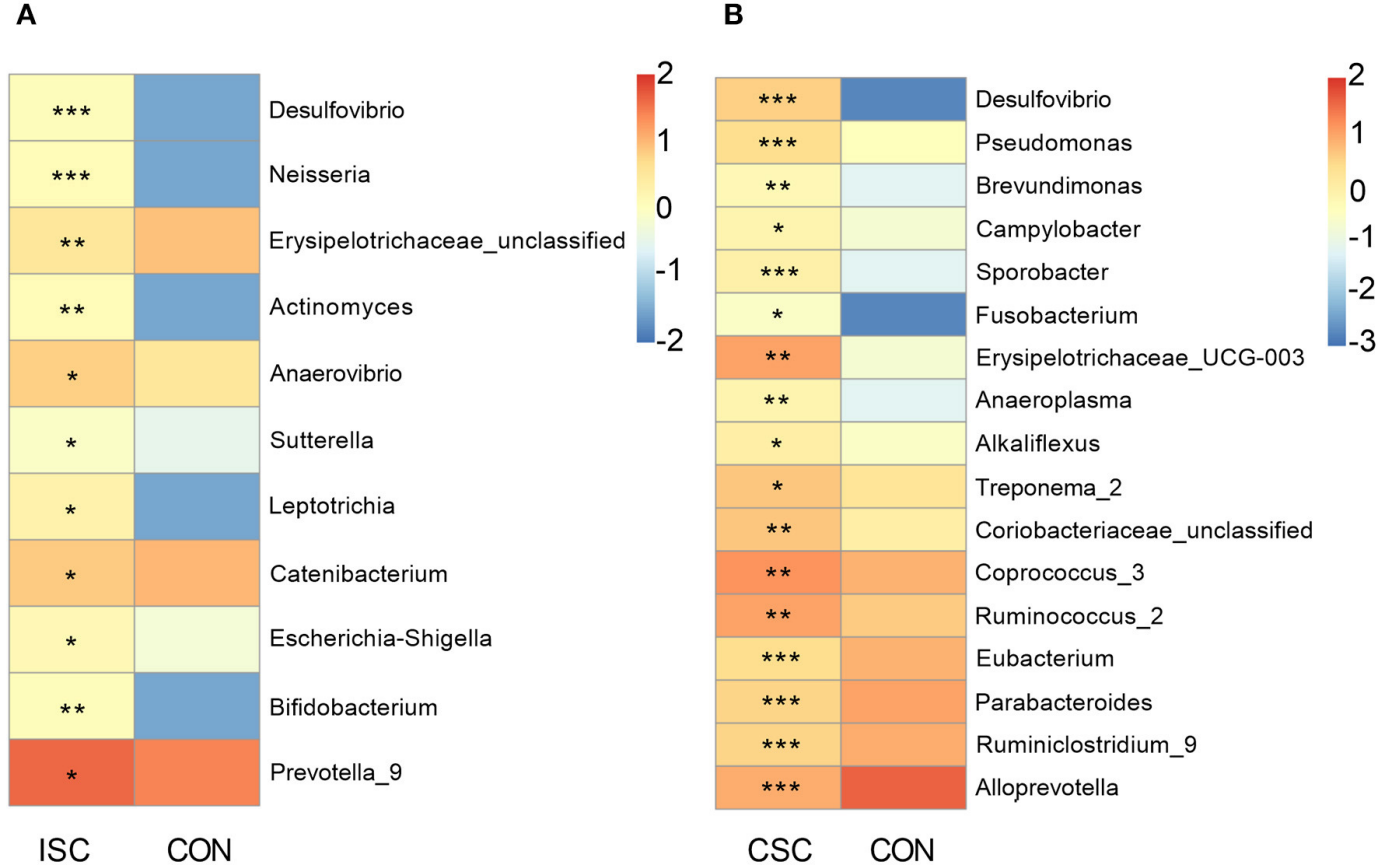
The abundance of differential genera in feces of piglets. **(A)** The differential genera between the ISC and CON groups. **(B)** The differential genera between the CSC and CON groups. The differential genera were analyzed by Wilcox test. All values are expressed as the means ± SEM. *p < 0.05, **p < 0.01, ***p < 0.001. ISC, intermittent social contact; CSC, continuous social contact; CON, control.

Compared with the CON group, the pernicious bacteria were significantly increased in both social contact groups (Figure 3). The abundances of pernicious bacteria (*Desulfovibrio, Neisseria, Erysipelotrichaceae_unclassified, Actinomyces, Anaerovibrio, Sutterella, Leptotrichia*, and *Escherichia-Shigella*) were significantly increased in the ISC group (*p* < 0.05). In contrast, the abundances of pernicious bacteria, including *Desulfovibrio, Pseudomonas, Brevundimonas, Campylobacter, Sporobacter, Fusobacterium, Erysipelotrichaceae_UCG-003, Anaeroplasma, Alkaliflexus*, and *Treponema_2* were significantly increased in the CSC group (*p* < 0.05) compared with the CON group. In addition, the abundance of beneficial bacteria, including *Bifidobacterium* and *Prevotella_9*, in the ISC group was significantly increased compared with the CON group (*p* < 0.05). The abundances of proinflammatory bacteria (*Coriobacteriaceae_unclassified, Coprococcus_3*, and *Ruminococcus_2*) were significantly increased (*p* < 0.05), but the abundances of anti-inflammatory bacteria (*Eubacterium, Parabacteroides, Ruminiclostridium_9*, and *Alloprevotella*) were significantly decreased in the CSC group (*p* < 0.05) compared with the CON group. The abundances of bacteria of ISC vs. CON and CSC vs. CON are shown in Supplementary Tables 5, 6.

### Correlation Analysis Between KEGG Pathways of DEGs and Differential Microbial Taxa

**Figure 4:**
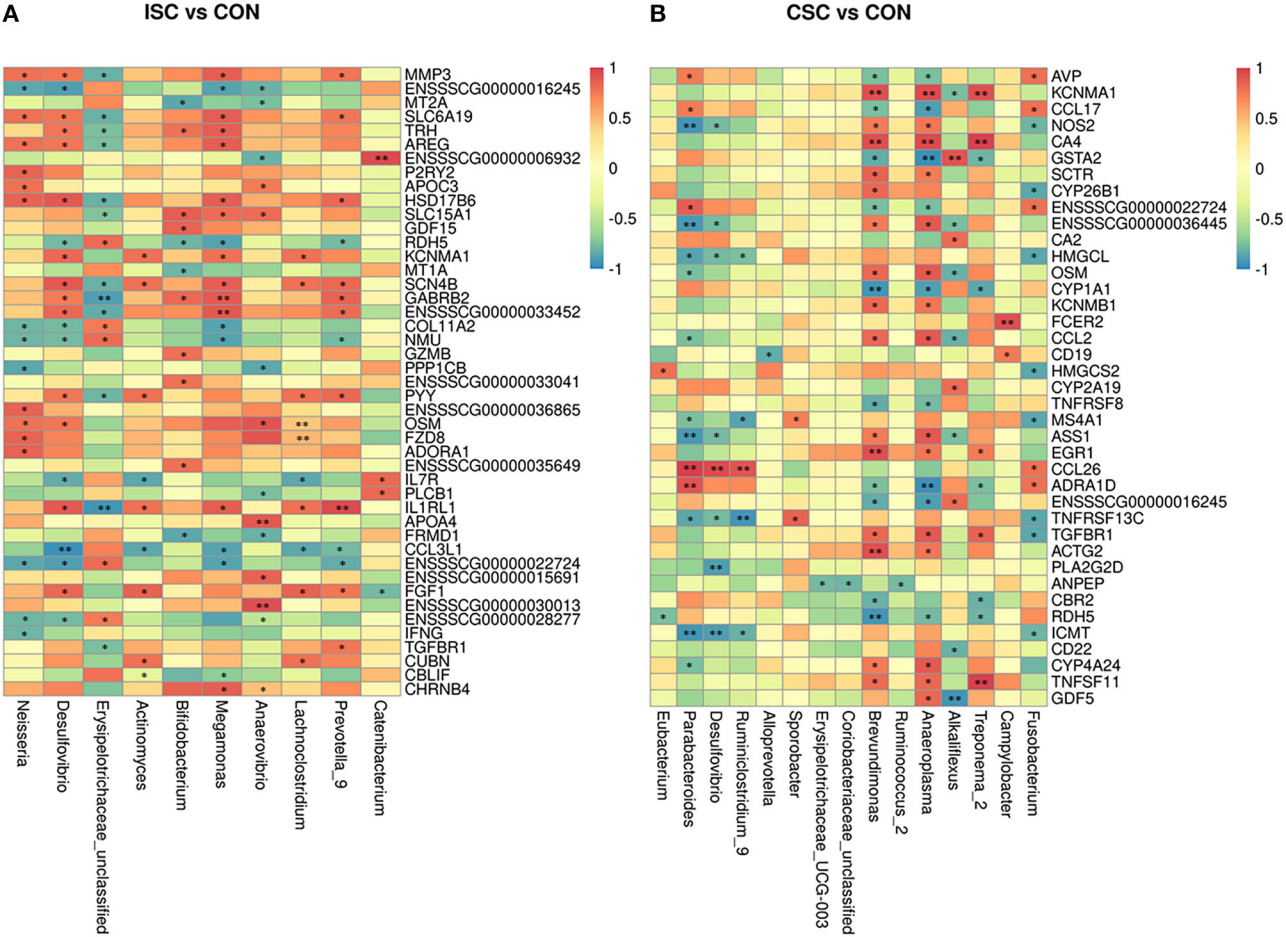
Heat map of Spearman's correlation coefficient between colonic differentially expressed genes and fecal microorganisms of piglets. **(A)** The correlation coefficient between the ISC and CON groups. **(B)** The correlation coefficient between the CSC and CON groups. *p < 0.05, **p < 0.01. ISC, intermittent social contact; CSC, continuous social contact; CON, control.

The correlation analysis between KEGG pathways of DEGs and differential microbial taxa between CSC and CON groups are shown in Figure 4. The correlation coefficient obtained by Spearman is shown in Supplementary Tables 7, 8. The correlation analysis of ISC and CON groups illustrated that *Bifidobacterium* was positively correlated with solute carrier family 15 member 1 (*SLC15A1*) but negatively correlated with metallothionein 1A (*MT1A*) and *MT2A*. Besides, *Megamonas* was positively correlated with solute carrier family 6 member 19 (*SLC6A19*), *SLC15A1*, and potassium calcium-activated channel subfamily M alpha 1 (*KCNMA1*) but negatively correlated with *Col11A2*. In addition, the correlation analysis of CSC and CON groups showed that *Brevundimonas* and *Treponema_2* were negatively correlated with *RDH5* and cytochrome P4501A1 (*CYP1A1*), whereas *CA4* was positively correlated with *Brevundimonas*.

### SCFAs Concentration of Colonic Contents

**Figure 5:**
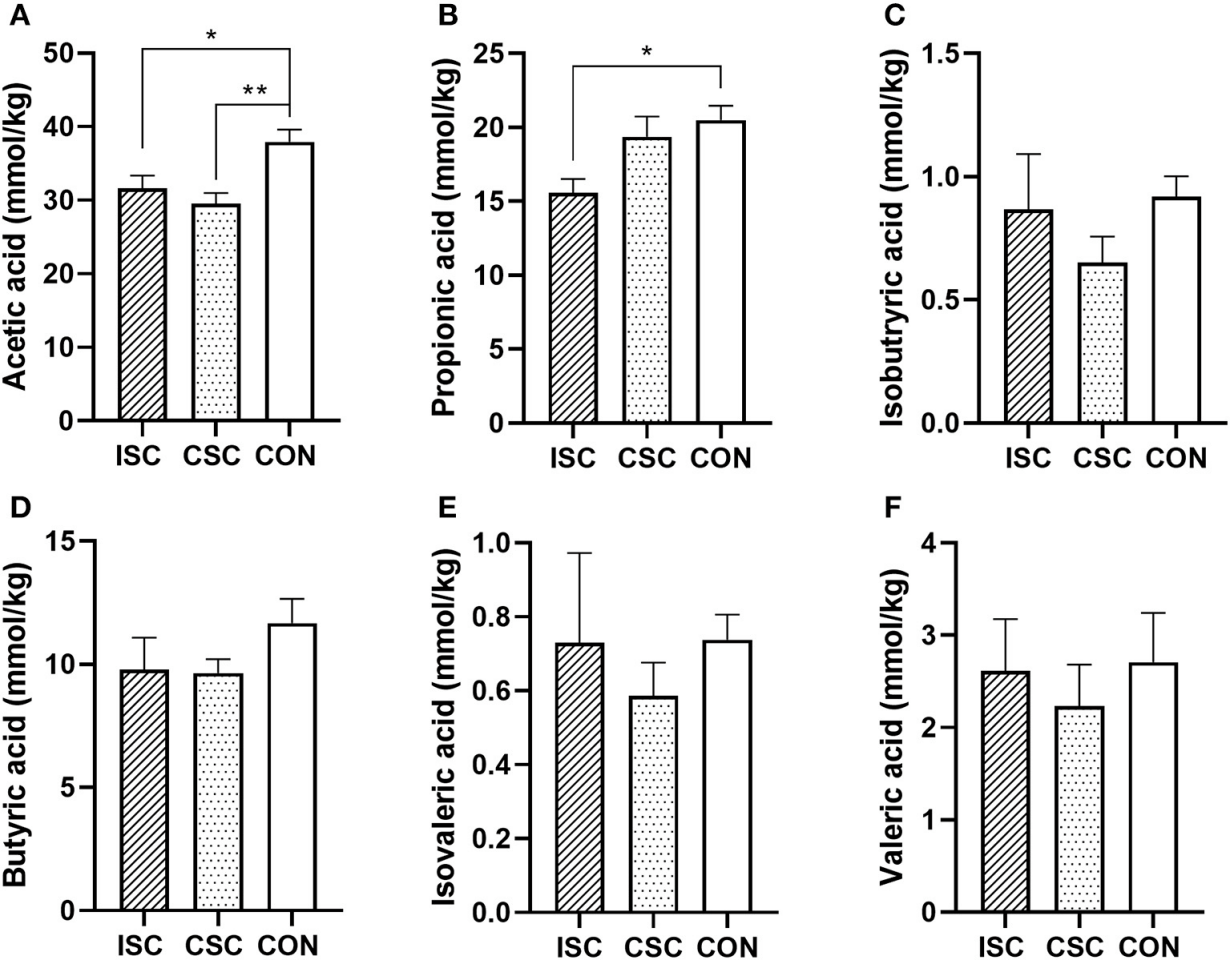
The concentration of SCFAs in the colonic content of piglets. **(A)** Acetic acid. **(B)** Propionic acid. **(C)** Isobutyric acid. **(D)** Butyric acid. **(E)** Isovaleric acid. **(F)** Valeric acid. *n* = 6, *p < 0.05 and **p < 0.01. SCFAs, short-chain fatty acids; ISC, intermittent social contact; CSC, continuous social contact; CON, control.

As shown in Figure 5, compared with the CON group, the concentrations of acetic acid (*p* = 0.026) and propionic acid (*p* = 0.028) in the ISC group were significantly decreased, and the concentration of acetic acid in the CSC group was significantly decreased (*p* = 0.004).

## Discussion

Piglets generally undergo three-time regrouping from birth to slaughter (Ko et al., 2020). Sudden regrouping results in aggression and abnormal behavior of piglets (O'Connell et al., 2005; Grümpel et al., 2018). Intensive rearing improves economic efficiency, which affects the welfare status of piglets (Martinez-Macipe et al., 2020). In the recent years, researchers provided enriched social environments for piglets in lactation to improve their welfare (Salazar et al., 2018; Ko et al., 2020). The lactating enriched social environment can reduce the weaning stress and aggression of piglets (Ko et al., 2020). In this study, we provided intermittent and continual social contact models during lactation, and our results showed that lactating social contact models altered the gene expression and microbial composition of piglets.

Proteins, minerals, and vitamins participate in the body's chemical reactions and maintain the body's internal environment (Danchin and Nikel, 2019; Lauridsen et al., 2021). The results of KEGG analysis on DEGs in this study showed that the ISC group mainly affected nutrient absorption and immune response of piglets. Compared with the CON group, *SLC6A19, SLC15A1*, cubilin (*CUBN*), apolipoprotein A4 (*APOA4*), and *KCNMA1* were significantly upregulated in the ISC group. *SLC6A19* and *SLC15A1* are the important neutral transporters in intestinal epithelial cells, which mediates transmembrane transport of amino acids and promotes amino acid absorption (Ganapathy and Leibach, 1983; Brer, 2008). *CUBN* has the function of reabsorbing fat-soluble vitamin carrier protein and also has a positive effect on the expression of vitamin B12 in the body (Aminoff et al., 1999). *APOA4* enhances the esterification and transport of vitamin A in intestinal epithelial cells (Hebiguchi et al., 2015). *KCNMA1* contributes to the potential polarization of cell membrane potential and induces the secretion of active K^+^ (Singh et al., 2012). The activity of K^+^ is important for maintaining cell membrane electrochemical potential and osmotic pressure (Danchin and Nikel, 2019). These upregulated genes had a positive effect on the digestion and absorption of proteins, minerals, and vitamins. Downregulated genes in this study included phospholipase C beta 1 (*PLCB1*), *MT1A, MT2A*, and glue prototype 11α chain (*COL11A2*). *PLCB1* contributes to wound healing and intestinal barrier recovery. A study found that *PLCB1* protein content in the injured intestinal contents of mice was significantly upregulated (Lee et al., 2013). *MT2A* promotes the upregulation of IFNA expression, and *MT1A* inhibits or reduces the production of reactive oxygen species (ROS) and the expression of proinflammatory factors in inflammatory enteritis to alleviate mucosal damage (Yamada et al., 2012). *Col11A2* is a marker for cancer (Roos et al., 2016) and inflammatory enteritis in humans (Dooley et al., 2004). The presence of these downregulated genes indicated that the intestinal status of piglets in the ISC group was relatively healthier than the CON group. In our previous study, ISC exposure during lactation had a positive effect on the digestion and absorption of protein, vitamins, and minerals and intestinal health of piglets, which also confirms the low FCR of piglets in the ISC group (Ji et al., 2021).

The effects of ISC and CSC on growing piglets were inconsistent. CSC mainly affected the nutritional metabolism of piglets. Retinol is necessary for the integrity and function of intestinal epithelial cells (Quadro et al., 2000). *RDH5* is one of the most important enzymes in retinol metabolism (Jette et al., 2004), and *CYP1A1* catalyzes retinol metabolism (Hoegberg et al., 2003). In our study, *RDH5* and *CYP1A1* were downregulated in the CSC group compared with the CON group, which suggested that CSC during lactation could cause retinol metabolism disorder, affecting the integrity of the colonic mucosal structure and function in piglets. Glutathione S-transferases (GSTs) participate in the release pathway of oxidative stress and detoxify exotic organisms, environmental carcinogens, and ROS (Il Gum et al., 2007). In this study, glutathione S-transferase A1-like (*GSTA2*) in the CSC group was significantly downregulated, indicating that piglets in the CSC group had a poor ability to relieve stress when exposed to the new environment. Carbonic anhydrase 2 (*CA2*) and carbonic anhydrase 4 (*CA4*) are involved in nitrogen metabolism and mainly participate in body acid-base balance and ion exchange. The overexpression or underexpression of carbonic anhydrase (CA) can easily disrupt the CA cycle, which induces an increase in the levels of NA^+^, Cl^−^, and water in the lumen, leading to diarrhea (Endeward et al., 2003; Khan and Khan, 2019). In our study, compared with the CON group, *CA4* was significantly upregulated, but *CA2* was significantly downregulated in the CSC group, which may cause CA cycle disorder and affect the health of piglets. Thus, CSC might reduce intestinal mucosal permeability and affect the intestinal nutrient absorption function of piglets.

The early living environment has a profound impact on animals (Guevarra et al., 2019), and social contact is one of the most important ways of microbial exchange in the host (Wikberg et al., 2020). Saladrigas-García et al. (2021) showed that higher bacterial diversity was related to mature intestinal microorganisms, which helps the body to maintain elasticity and stability under environmental stress. In this study, intestinal microbial diversity indexes, Chao1, and observed_species were increased in piglets in the ISC group, which suggests that the intermittent social contact model may cause microbial exchange among piglets, stimulating the development and maturity of intestinal microorganisms and helping the organism to adapt to the new environment. However, there was no difference in bacterial diversity between the CSC and CON groups, indicating that continuous social contact during lactation did not affect bacterial diversity in the feces of growing piglets.

Consistent with the findings of Saladrigas-García et al. (2021), the composition of intestinal microorganisms of piglets was influenced by social contact during lactation in this study. Firmicutes and Bacteroides were dominant phyla, but their abundance was not different among groups. However, compared with the CON group, the abundances of pernicious bacteria (*Desulfovibrio, Pseudomonas, Brevundimonas, Campylobacter, Sporobacter, Erysipelotrichaceae_UCG-003, Anaeroplasma, Alkaliflexus, Treponema_2*, and *Fusobacterium*) were significantly increased in the CSC group at the genus level. The abundance of *Desulfovibrio* was increased in the cecum of rats with the increase in intestinal permeability (Qu et al., 2017) and proinflammatory factor, interleukin 2 (IL-12) in the cecal lymph nodes of horses (Lindenberg et al., 2019). *Sporobacter* is negatively correlated with the body weight of mice (He et al., 2016). Tan et al. (2018) reported that the abundance of *Anaeroplasma* was lower in the ileum of pigs with high feed digestibility and was also higher in rex rabbits with low body weight (Zeng et al., 2015). Our previous study found that piglets reared in the CSC group had less bodyweight gain than those in the CON group (Ji et al., 2021), which might suggest that CSC impaired bodyweight of piglets by increasing the abundance of *Anaeroplasma*. It is known that *Brevundimonas* and *Treponema_2* are pathogenic bacteria. This study found that the expression levels of *RDH5* and *CYP1A1* genes related to intestinal mucosal protection in the colonic mucosa were decreased, and *RDH5* and *CYP1A1* were negatively correlated with *Brevundimonas* and *Treponema_2*. Thus, increased *Brevundimonas* and *Treponema_2* might inhibit *RDH5* and *CYP1A1* expression impairing the health of colonic mucosa of piglets in CSC group. The upregulation of these pernicious microorganisms in the feces of piglets from the CSC group may affect intestinal permeability and reduce the weight gain of piglets. Compared with the CON group, the abundances of *Lachnospiraceae_UCG-010, Parabacteroides, Anaerotruncus, Faecalicatena, Prevotella, Prevotella_2*, and *Faecalibacterium* in the CSC group were significantly downregulated, but those of *Lachnospiraceae_XPB1014_group, Lachnospiraceae_AC2044_group, Lachnospiraceae_NK4B4_group, Lachnospiraceae_NK3A20_group, Lachnospiraceae_NK3A20_group, Gastranaerophilales_unclassified*, and *Coprococcus_3* were upregulated. These bacteria are related to the production of SCFAs (Sasaki and Klapproth, 2012; Zhai et al., 2017; Zhao et al., 2017). Thus, continual social contact could influence SCFAs production. The abundance of *Parabacteroides* was decreased in the inflammatory intestine of mice induced by polyethylene microplastics (Li et al., 2019). *Prevotella* treats and prevents diarrhea of piglets (Dadi et al., 2020) and is negatively correlated with coding proinflammatory factor, forkhead box P3 in colonic lymph nodes (Lindenberg et al., 2019). *Prevotella_2* is beneficial to feed intake, feed efficiency, fat accumulation, and muscle growth (Qi et al., 2021). *Faecalibacterium* is the main producer of butyric acid in the intestine (Sasaki and Klapproth, 2012), which is the main energy source of the colon. Besides, *Coprococcus_3* is positively correlated with the disorder of lipid metabolism and proinflammatory response in the ileum of low-birth weight piglets (Huang et al., 2020). These results showed that the SCFA-producing bacteria were affected by CSC, among which the increased microbes were harmful to the host, but the decreased microbes were beneficial. CSC during lactation increases the abundance of pernicious and proinflammatory bacteria and decreases the anti-inflammatory bacteria, which may cause lower feed efficiency in the CSC group.

Compared with the CON group, the abundances of pernicious microorganisms (*Desulfovibrio, Neisseria, Sutterella*, etc.) and anti-inflammatory bacteria (*Megamonas, Lachnoclostridium*, and *Bifidobacterium*) were significantly upregulated in the ISC group. A study reported that *Bifidobacterium* could maintain the proper balance of bacteria, reduce the risk of pathogen infection, and prevent pernicious bacteria from colonizing and invading the host (Gaggìa et al., 2010). Otherwise, *Bifidobacterium* also has a negative effect on *MT1A* and *MT2A* that promote the expression of proinflammatory factors (Yamada et al., 2012), suggesting that *Bifidobacterium* could protect the gut from proinflammatory factors. *Megamonas* can resist *Campylobacter* of chicken (Hertogs et al., 2021) and has a positive effect on *SLC6A19, SLC15A1*, and *KCNMA1* expression in this study, which might illustrate that *Megamonas* could maintain colonic health by regulating these genes' expression. Butyrate-producing *Lachnoclostridium* can alleviate enteritis (Zhao et al., 2017). Although the abundance of certain pernicious bacteria was increased in the ISC group, the increased anti-inflammatory and beneficial bacteria could prevent several pernicious bacteria from invading the body through the intestinal mucosa. Thus, piglets in the ISC group kept a relatively healthy intestinal tract. In addition, both pernicious and beneficial bacteria were also increased in the feces of the ISC group in this study, and our recent study found that ISC did not affect the growth performance but improved the feed efficiency of piglets (Ji et al., 2021). We speculated that slight adverse effects induced by the intermittent social contact model in early life might stimulate the intestinal growth and development of piglets. Overall, the abundances of pernicious bacteria were increased in both ISC and CSC groups, whereas the abundances of beneficial bacteria were increased only in the feces of piglets in the ISC group.

Short-chain fatty acids are the main products of anaerobic microbial fermentation of dietary fiber in the colon (Luu et al., 2019). SCFAs provide energy for intestinal epithelial cells, affect intestinal peristalsis, maintain intestinal barrier function and host metabolism, and regulate intestinal immunity (Donohoe et al., 2011; Wu et al., 2016). SCFAs can provide 5–25% of energy for pig growth (Stumpff, 2018). The acetate is a substrate for cholesterol and fatty acid synthesis (Boets et al., 2017). Our previous studies showed that the CSC group's piglets had a higher FCR and lower body weight than the CON group's piglets, but there was no significant difference in the daily weight gain and FCR between the ISC and CON groups (Bi et al., 2021; Ji et al., 2021). Although the concentration of acetate was downregulated in both ISC and the CSC groups compared with the CON group in this study, the growth performance of piglets was not consistent among groups, which may indicate the changes in microbial composition and abundance. The expression levels of colonic genes related to nutrient absorption affected the utilization of acetate, influencing the growth of piglets. Psichas et al. (2015) found that propionate could stimulate the release of *PYY* that suppress the host's appetite. This study showed that the concentration of propionate was decreased, and *PYY* was upregulated in the ISC group compared with the CON group. Also, the ISC group's piglets had a lower feed intake compared with the CON group's piglets (average daily feed intake per growing piglets: ISC: 0.61 ± 0.01 kg; CON: 0.71 ± 0.01, *p* = 0.01), but there was no difference in propionate and *PYY* between both CSC and CON groups. It may suggest that ISC can impair the appetite, and the feed intake of piglets is reliable by directly stimulating *PYY* expression. Both intermittent and continuous social contact models during lactation decreased the concentrations of acetate and propionate in the colonic content of piglets, which may be influenced by the differential microbes. But the changed concentrations of acetate and propionate had limited effects on the growth performance of piglets in this study. Thus, we need further experiments to understand the underlying mechanisms of different social contact models on the absorption capacity of SCFAs in piglets.

## Conclusion

In this study, the abundances of pernicious microorganisms in the feces of piglets were increased in ISC and CSC groups, and the abundances of beneficial bacteria were increased in the feces of the CSC group, which may lead to the imbalance of colon ion metabolism and affect the absorption of nutrients. Although the abundance of pernicious bacteria was increased in the ISC group, the abundances of *Bifidobacterium* and *Prevotella_9* were also synchronously increased. This study suggested that different social contacts during lactation altered intestinal microbe and colonic nutrient absorption related-gene expression in piglets, but intermittent social contact had positive impacts on the fecal composition and intestinal health of growing piglets.

## Data Availability Statement

The datasets presented in this study can be found in online repositories. The names of the repository/repositories and accession number(s) can be found at: NCBI BioProject—PRJNA668937, PRJNA771754, PRJNA757700, and PRJNA786659.

## Ethics Statement

The animal study was reviewed and approved by Animal Care and Use Committee of Northeast Agricultural University NEAU-[2011]-9.

## Author Contributions

YB, HL, and JB contributed to the conception and design of the study. YB and HW wrote the first draft of the manuscript and performed the statistical analysis. YB, HN, RL, WJ, and HW were involved in the animal experiments and data collection. All authors read and approved the final manuscript.

## Funding

The study was supported by Earmarked Fund for China Agriculture Research System of MOF and MARA (CARS-35-05B).

## Conflict of Interest

The authors declare that the research was conducted in the absence of any commercial or financial relationships that could be construed as a potential conflict of interest.

## Publisher's Note

All claims expressed in this article are solely those of the authors and do not necessarily represent those of their affiliated organizations, or those of the publisher, the editors and the reviewers. Any product that may be evaluated in this article, or claim that may be made by its manufacturer, is not guaranteed or endorsed by the publisher.
